# A 13‐year‐old soccer player presenting with a Morel‐Lavallee lesion of the knee: A case report and review of literature

**DOI:** 10.1002/ccr3.9365

**Published:** 2024-08-27

**Authors:** Nicholas R. Williams, Alvarho J. Guzman, Patrick Waldron, Ethan Vallelanes, Samantha J. Cheng, James L. Chen

**Affiliations:** ^1^ Advanced Orthopedics and Sports Medicine San Francisco California USA

**Keywords:** Morel‐Lavallee lesion, orthopedics, pediatrics and adolescent medicine, radiology and imaging, sports medicine

## Abstract

Morel‐Lavallee lesions are uncommon injuries and there is a lack of data to support what the most effective management is. In the case of a young athlete with a small lesion, we propose that conservative treatment with rest and close follow up is appropriate.

## Graphical abstract

Subcutaneous fluid collection in a 13‐year‐old soccer player.

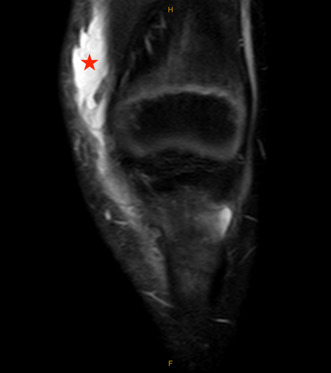

## 1 INTRODUCTION

Morel‐Lavallée Lesions (MLL) are an uncommon type of closed, traumatic degloving injury that can occur among patients with polytrauma.^1^ The first case of MLL was reported in 1863 by a French surgeon named Victor‐Auguste‐François Morel‐Lavallée in a 60‐year‐old woman who sustained a motor vehicle collision (MVC).^2^ MLL result from shearing forces that cause the superficial subcutaneous tissue to separate from the underlying fascial layer, which results in the formation of a cavity.^3^ Damage to the trans‐aponeurotic capillaries and lymphatic vessels causes blood and lymph to leak into the developed cavity.^3^ Gradually, the cavity will resolve on its own as the blood and fluid are reabsorbed. However, the hemosiderin layer that surrounds the remaining serosanguinous fluid may cause inflammation within the peripheral tissues, resulting in a fibrous capsule that stops reabsorption and causes chronic MLL.^3^

MLL are difficult to diagnose in the acute setting because they are commonly associated with high‐impact traumatic injuries and are easily overlooked as subcutaneous hematomas during primary and secondary surveys.^4^ Most lesions are identified during routine surgical intervention, commonly such as open reduction and internal fixation (ORIF) of a fracture. With high clinical suspicion, imaging modalities such as ultrasound and magnetic resonance imaging (MRI) can be implemented to aid in the diagnosis of these lesions. MRI is the gold standard for diagnosis but may be substituted with ultrasound if clinical suspicion of MLL is high. Both imaging modalities will demonstrate a subcutaneous fluid collection that is typically extra‐articular and well‐encapsulated.

Treatment options include both operative and nonoperative management. Nonoperative therapies include aspiration, compression, soft tissue manual therapy, dry needling with electrical stimulation, corrective exercises, and radial extracorporeal shockwave therapy.^5^ Surgical management consists of sclerotherapy, percutaneous or open debridement, and dead space closure. Surgical planning depends heavily on the presence of associated fractures and whether or not they are open or closed. Both immediate and staged fixation have been described with varying success, but surgical debridement before internal fixation is necessary to avoid postoperative hematoma.^1^ When electing for staged treatment, external fixator pins through the lesion should be avoided if possible.^1^

Treatment protocols for MLL have not been well defined due to a paucity of reported cases. Typically, clinical judgment has been used to guide management, but Singh et al.^3^ recently published an algorithm for the management of MLL based on a meta‐analysis of 17 studies. They proposed that acute lesions should be managed with doxycycline sclerosis, or compression bandaging if the lesion is over the knee. Chronic lesions >400 mL should be treated with open drainage or mass resection, and those <400 mL should be treated with doxycycline sclerodesis. While several other algorithms have also been proposed in the literature, there are still no established guidelines for management. This is especially true for the adolescent population and MLL sustained from participation in sports, for which treatment protocol is typically tailored on a case‐by‐case basis.

## 2 CASE HISTORY

A 13‐year‐old Caucasian male presented to our clinic with left knee pain, 1 week after suffering an injury while playing soccer. He reports that he was tripped by another player and landed on the ground with his left knee. At the time of the injury, he had immediate pain and swelling and was unable to bear weight. He went to a local emergency department for an initial evaluation. There, radiographs of the left knee demonstrated medial soft tissue swelling without any fractures or dislocations. He was placed in a T‐scope brace and recommended to follow up with an orthopedic surgeon.

**FIGURE 1:**
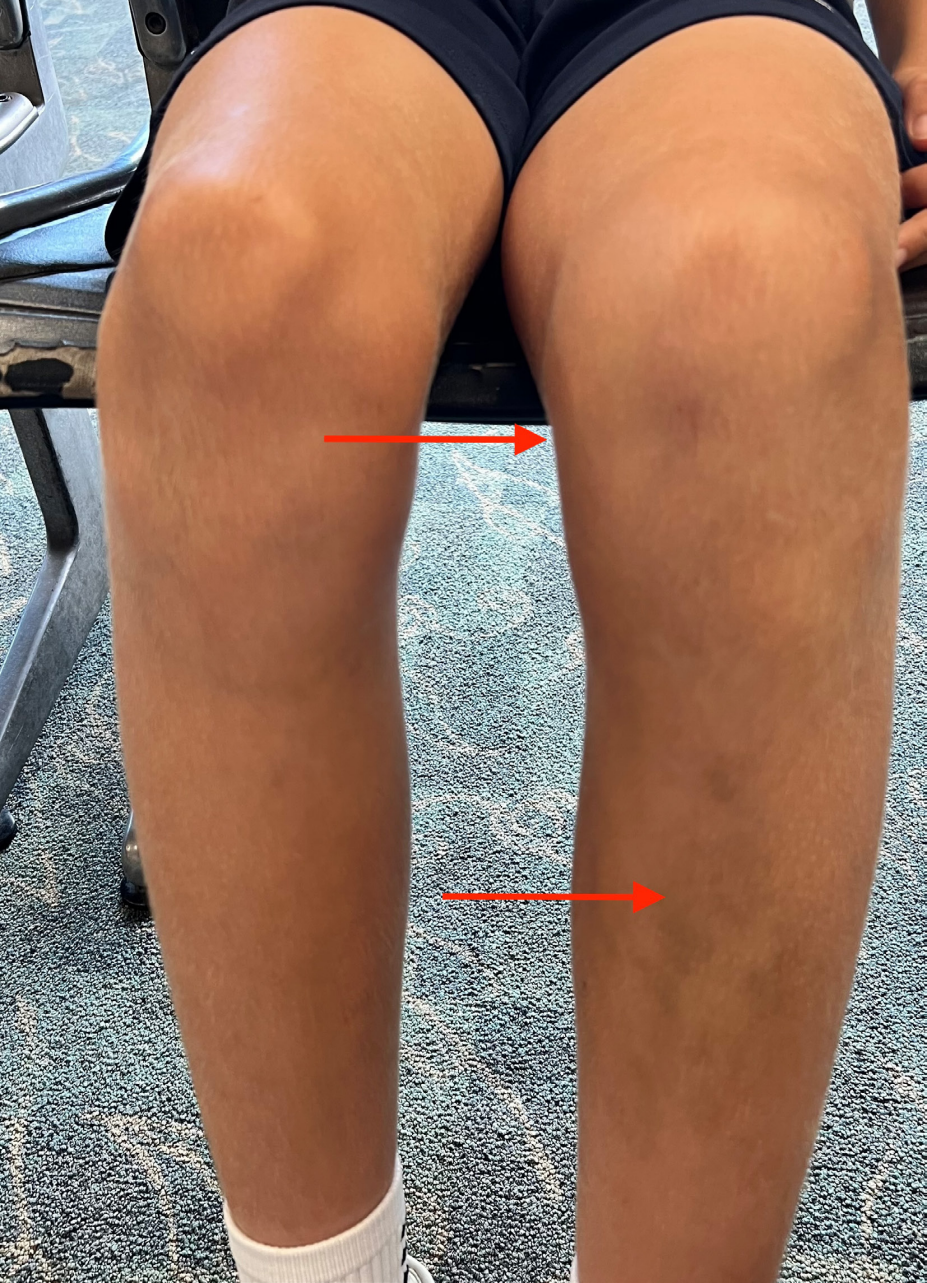
72 h postinjury; Image of the bilateral knees approximately 72 h following initial injury which demonstrates ecchymosis, edema, and effusion in the left knee compared to the contralateral side.

Upon evaluation in our clinic, the patient presented full weight bearing in a T‐scope brace with an antalgic gait. He reported difficulty with extension of the left knee so he was using a toe‐walking gait pattern. Further examination of the left knee demonstrated extensive ecchymosis about the suprapatellar, medial, and proximal anterior tibial areas with an associated suprapatellar effusion (Figure 1). He had tenderness to palpation at the medial joint line and the anterior patella. Patellar apprehension test was negative. Range of motion was from 10 to 80 degrees with pain at the extremes of flexion and extension. Lachman's test was negative. There was no laxity with varus or valgus stress. He was neurovascularly intact.

## 3 METHODS

**FIGURE 2:**
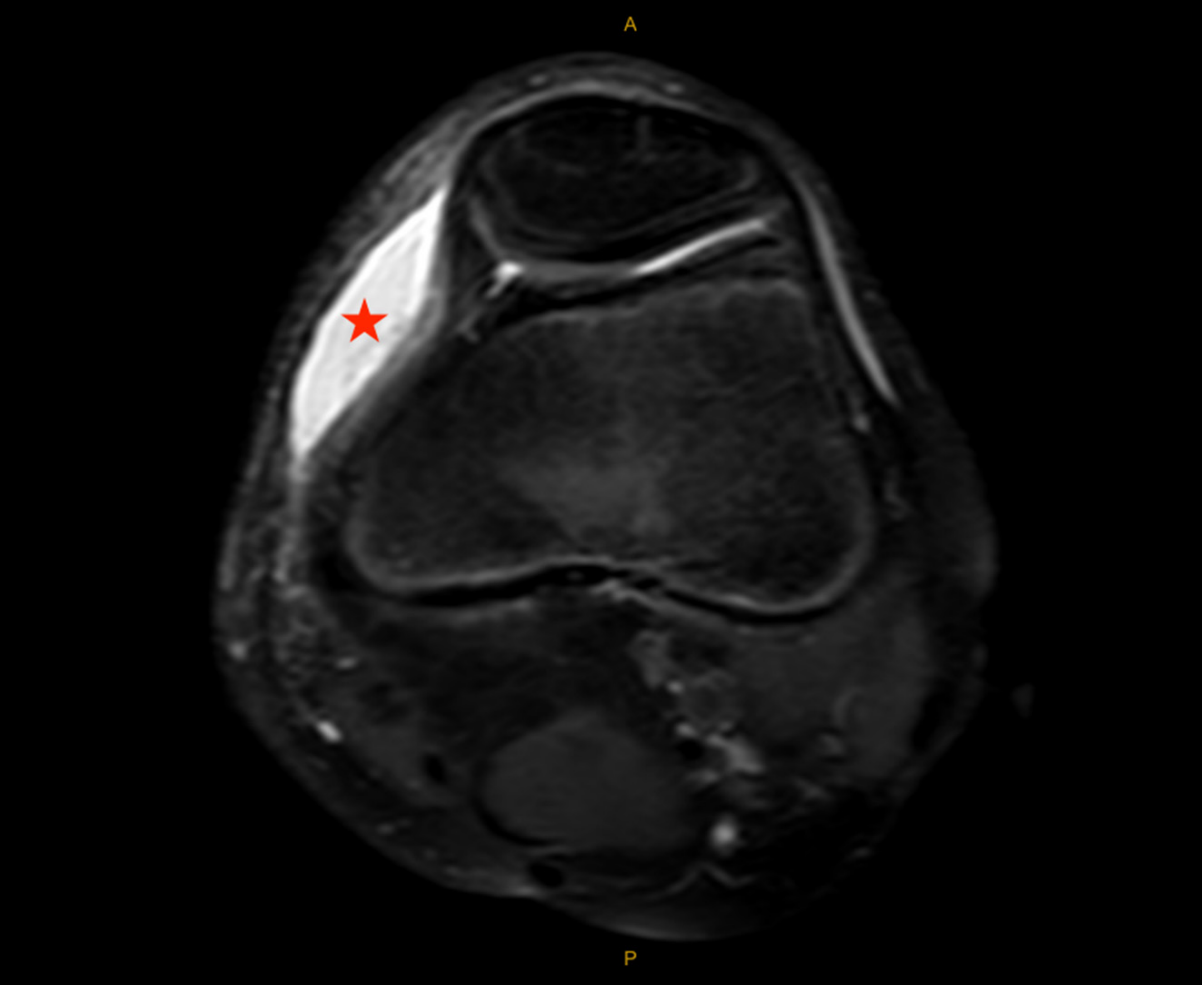
Axial MRI; Proton density fat‐saturation axial MRI sequence of the left knee obtained 8 days after the initial injury showing a subcutaneous fluid collection (star) at the anteromedial aspect of the knee, measuring about 4 cm anterior–posterior.

**FIGURE 3:**
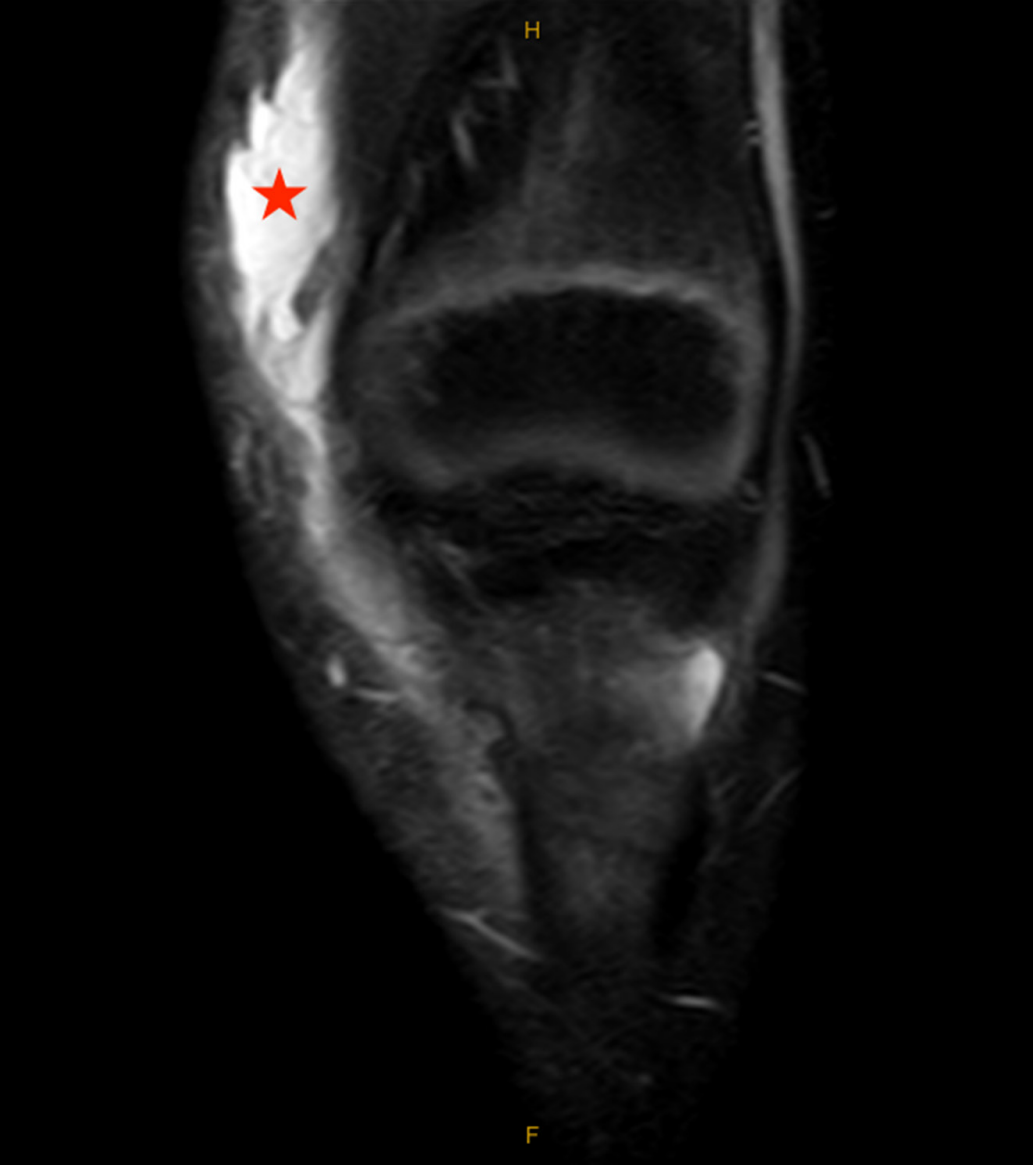
Coronal MRI; Proton density fat‐saturation coronal MRI sequence of the left knee obtained 8 days after the initial injury showing a subcutaneous fluid collection (star) at the anteromedial aspect of the knee, measuring about 5 cm craniocaudal.

**FIGURE 4:**
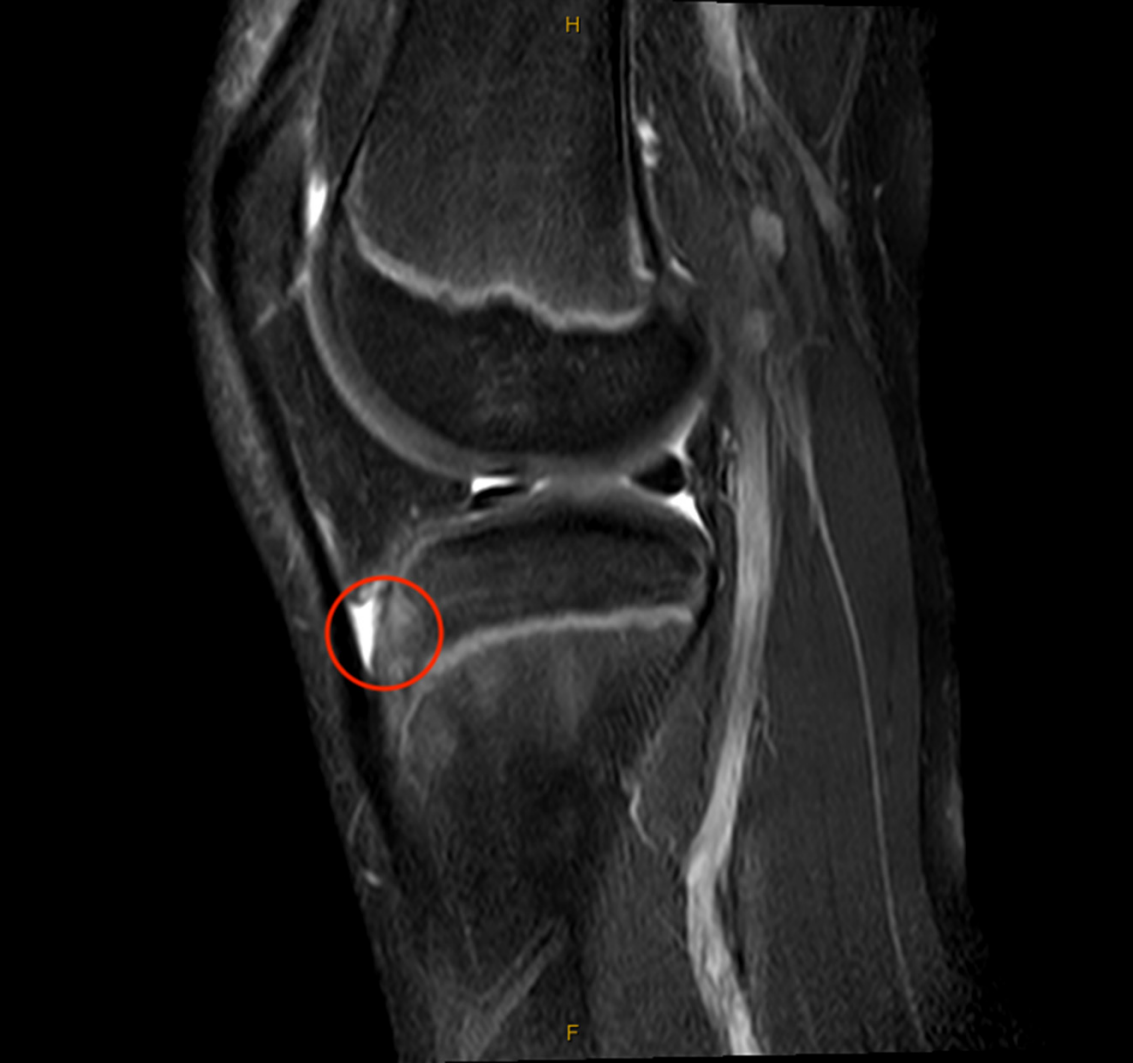
Sagittal MRI; Proton density fat‐saturation sagittal MRI sequence of the left knee obtained 8 days after the initial injury showing mild bone marrow edema in the tibial tubercle and a small amount of reactive fluid in the adjacent deep infrapatellar bursa (circle).

Based on the patient's history and physical examination, the decision was made to obtain magnetic resonance imaging (MRI) of the left knee without contrast. MRI obtained 8 days after the injury demonstrated an elliptical subcutaneous fluid collection at the anteromedial aspect of the knee located superficial to the medial patellofemoral ligament (MPFL), medial patellar retinaculum, and vastus medialis oblique (VMO) muscle measuring 5 × 4 × 1 cm (craniocaudal (CC), anteroposterior (AP) oblique, transverse (TR) oblique) with surrounding soft tissue edema (Figures 2 and 3). The tibial tubercle had mild bone marrow edema consistent with contusion, and the adjacent deep infrapatellar bursa had a small amount of reactive fluid (Figure 4). There were no ligamentous or meniscal injuries noted. There was no patellofemoral chondral defect or findings of transient lateral patellar dislocation.

## 4 CONCLUSION AND RESULTS

These results were communicated to the patient and his parents. Based on the patient's age and the size of the lesion the decision was made to allow the lesion to heal on its own with close follow‐up. Anticipatory guidance was given concerning potential signs of infection to look out for. One month after the injury he began physical therapy to return to athletics. No follow‐up imaging was obtained to assess the change in size of his lesion. Clinically he did not have any associated erythema, ecchymosis, or edema, and he had full range of motion without pain.

## 5 DISCUSSION

MLL are characterized by closed, internal degloving of subcutaneous tissues resulting primarily through high‐energy fractures or crush injuries, as well as low‐energy sports injuries. MLL are rare in nature but tend to occur most commonly at the hip, thigh, or knee. Tseng et al.^6^ reported that among roughly 1100 patients from 1994 to 2004 who presented to Boston University Medical Center with pelvic and acetabular fractures, only 19 were found to have MLL. Nickerson et al.^7^ identified 79 patients with 87 MLL in the setting of trauma. They found that 49 out of 87 (56%) of MLL were associated with high‐energy mechanisms, whereas 30 out of 87 (34%) of MLL were associated with fractures. Various different treatment algorithms^1^, ^3^, ^8^ have been proposed for the management of MLL, but there is not one widely agreed upon approach reported in the literature.

In addition to being associated with high‐energy trauma, MLL are also known to have a high risk of infection. Hak et al.^9^ retrospectively looked at a series of 24 MLL and found that initial cultures were positive in 46% of cases. The incidence of positive cultures was not dependent on the time from injury to debridement. There is still debate about the most effective management of MLL resulting from high‐energy mechanisms; however, conservative treatment is only recommended if the MLL is distant from skeletal structures, due to the lower risk of deep soft‐tissue and bone infections.^10^

The first point of contention is whether conservative or invasive treatment is more appropriate. Moriarty et al.^11^ identified a series of 66 MLL among 63 patients and investigated the follow‐up size of the lesion with conservative versus invasive treatment. Management data were documented in 59% (*n* = 39) of lesions. Of these, 33.3% (*n* = 13) had noninvasive treatment with external compression alone, while 66.6% (*n* = 26) had invasive treatment including aspiration, drain insertion, or washout. They found that at a mean follow‐up duration of 3.4 months, there was no significant difference in lesion size for those who had invasive compared to conservative treatment.

Conversely, Shen et al.^12^ suggested that surgical treatment leads to significantly better outcomes than conservative treatment. They identified a total of 21 studies published between 1997 and 2012 detailing the cases of 153 patients with MLL in the peri‐pelvic region. In total, 13 patients underwent conservative treatment and had a healing rate of less than 50%, compared to 133 of the 140 patients who underwent various types of surgical interventions. However, the size of the lesions were not documented, limiting the utility of these results. Nickerson et al.^7^ found that aspiration of more than 50 mL of fluid from MLL was much more common among lesions that recurred (83%) than among those that resolved (33%). Therefore, they proposed the practice management guideline that aspiration of more than 50 mL of fluid from an MLL should prompt operative intervention.

One of the newer methods of treatment which has shown promising results thus far is doxycycline sclerodesis. Bansal et al.^13^ described a series of 16 persistent MLL that were successfully treated with doxycycline‐induced sclerotherapy and compression bandaging. The mean duration of MLL persistence was 13 months, with an average of 3.44 previous aspirations prior to sclerodesis. Eleven patients had complete resolution of symptoms at 4 weeks, and an additional four patients had resolution of symptoms at 8 weeks. The remaining patient was noncompliant with compression bandaging and their lesion persisted at 12 weeks, but was subsequently resolved on repeating the procedure.

While MLL commonly result from traumatic, high‐impact mechanisms, they are also reported in individuals playing sports as well. Khodaee et al.^14^ specifically investigated MLL that were identified in athletes. They identified 14 published case reports containing 45 athletes who were found to have MLL. They reported that 73% (*n* = 33) were the result of direct contact with the field, while 16% (*n* = 7) were the result of direct contact with another athlete.

There are few reported cases in the literature about MLL of the knee in adolescent athletes. Divjak et al.^15^ described two such cases. In the first, a 10‐year‐old soccer player was treated with needle aspiration of 25 mL of fluid and compression and was asymptomatic at 2‐week follow‐up. In the second, a 16‐year‐old skier was treated with needle aspiration of 85 mL of fluid and compression and was asymptomatic at 4‐week follow‐up. Vess et al.^16^ reported the case of a 14‐year‐old football player who was treated with needle aspiration of 26 mL of fluid and compression and was asymptomatic at one‐week follow‐up. Lastly, Depaoli et al.^17^ highlighted an 18‐year‐old soccer player who was treated conservatively with rest alone. There was no effusion noted on sonographic exam at 3‐month follow up.

The management of MLL resulting from low‐energy sports mechanisms differs widely from the management of MLL in high‐energy polytrauma patients. In general, they are much smaller in size, ranging from 25 to 85 mL in the studies above, compared to the mean of 181.9 mL found by Moriarty et al.^11^ As such, generally a more conservative approach is preferred. While both rest alone and needle aspiration with compression have been shown to be effective, the recovery time with needle aspiration and compression may be shorter than rest alone based on this limited data. Further investigation is necessary to determine the best treatment approach for athletes with small MLL.

This case report demonstrates a MLL sustained in a 13‐year‐old male soccer player that was successfully managed through observation alone. MLL of the knee can have potentially devastating effects on adolescents, a population with rapidly developing musculoskeletal systems and unique psychosocial demands, such as high pressure to return to play. However, in young patients with small (<50 mL) lesions, it is possible to observe the patient closely and allow the body time to resorb the fluid collection naturally. Small lesions do not require urgent intervention, but aspiration is often taken into consideration to mitigate future complications, especially in acute cases.

The decision to proceed with simple observation was influenced by several factors indicating a low risk for recurrence or complications. The patient presented with a relatively small lesion size (~20 mL of fluid approximated by cavity dimensions on MRI), and the patient's young age further supported noninvasive management. Aspiration and compression have previously been shown to be efficacious for the treatment of MLL of the knee in athletes,^15^, ^16^, ^18^ while our case details that observation alone can be a viable alternative in young patients with small fluid collections and minimal functional impairment. Simple observation promotes natural healing processes and minimizes the risks of more invasive treatments. However, additional research is needed to compare conservative versus minimally invasive treatments in the management of MLL in adolescent athletes.

## AUTHOR CONTRIBUTIONS

**Nicholas R. Williams:** Investigation; writing – original draft. **Alvarho J. Guzman:** Writing – original draft. **Patrick Waldron:** Writing – original draft. **Ethan Vallelanes:** Writing – original draft. **Samantha J. Cheng:** Writing – original draft. **James L. Chen:** Supervision.

## FUNDING INFORMATION

There are no funders to report for this submission.

## CONSENT

Written consent was obtained through the patient's mother to publish this case report with all identifying information removed.

## Data Availability

Data sharing not applicable to this article as no datasets were generated or analysed during the current study.
